# Temporal muscle thickness has no prognostic relevance in patients with high-grade glioma compared to functional scales

**DOI:** 10.3389/fonc.2023.1237105

**Published:** 2023-08-31

**Authors:** Julia Klingenschmid, Aleksandrs Krigers, Victoria Schön, Daniel Pinggera, Johannes Kerschbaumer, Astrid E. Grams, Claudius Thomé, Christian F. Freyschlag

**Affiliations:** ^1^ Department of Neurosurgery, Medical University of Innsbruck, Innsbruck, Austria; ^2^ Department of Radiology, Medical University of Innsbruck, Innsbruck, Austria

**Keywords:** frailty, temporal muscle thickness, GBM, CFS, survival

## Abstract

**Background:**

GBM research is constantly assessing potential valuable prognostic biomarkers to better understand the disease and prognosticate future outcomes. Measuring temporal muscle thickness (TMT) has appeared to be a promising new surrogate marker for skeletal muscle mass and sarcopenia, which further indicates frailty and predicts overall survival (OS). The aim of this study was to determine its usefulness as a prognostic marker in patients with high-grade glioma compared to functional status scales.

**Methods:**

TMT was measured in preoperative axial T1-weighted contrast-enhanced magnetic resonance images in 277 patients who received surgical treatment of newly diagnosed WHO III and IV gliomas in our institution between 2015 and 2020. Clinical Frailty Scale (CFS) and Karnofsky Performance Scale (KPS) were assessed preoperatively and during a follow-up visit.

**Results:**

Female gender has shown significant correlation with TMT, while TMT did not correlate with preoperative and follow-up functional scores, age, WHO classification, IDH mutation, MGMT promoter methylation, EGFR and ATRX expression, or 1p/19q co-deletion. No significant prognostic value of TMT could be shown in 6, 12, and 24 months OS, while changes in CFS and KPS proved to have a significant impact.

**Conclusion:**

Only female gender, but no other clinical, histological, or molecular marker showed any interrelation with TMT. Functional scores outclass measuring TMT as a reliable prognostic factor for predicting OS in patients with high-grade glioma.

## Introduction

Frailty is a relevant prognostic factor in patients with high-grade glioma and brain metastases, resulting in significant worsening of functionality and shorter overall survival (OS) in frail patients (1, 2). These patients can be identified using a variety of scoring systems. Karnofsky Performance Status Scale (KPS) is a tool that has been routinely used in neuro-oncology to determine suitability for chemotherapy for decades (3). The Clinical Frailty Scale (CFS) was developed by Rockwood et al. and scores the patients from 1 (very fit) to 9 (terminally ill) points (4). In comparison to KPS, it allows one to assess patients independently including a variety of patient’s restrictions regarding physical and mental health. CFS showed superior prediction of OS in patients with high-grade glioma and brain metastases in relation to KPS (1, 2).

One of the conditions, in which the complex syndrome of frailty usually manifests, is sarcopenia (5, 6). Since 2017, it has been listed in the International Statistical Classification of Diseases and Related Health Problems (ICD-10) as M62.84 (7) and is primarily defined by diminished muscle strength (8). Signs and symptoms of sarcopenia could be falling, feeling weak, rising from a chair with difficulty, walking more slowly, and losing weight unintentionally (9, 10). Magnetic resonance imaging (MRI) is the gold standard for the visualization and morphological quantification of the skeletal muscle mass (SMM) (11). Dual energy x-ray absorptiometry (DXA) (12) and bioelectrical impedance analysis (BIA) (13) are other options to calculate muscle quantity. Numerous physical tests including the Short Physical Performance Battery (SPPB), Timed-up and Go test (TUG), and measuring gait speed can be useful to evaluate frailty by assessing patients’ physical performance (8). Hence, characteristics that have been included to determine frailty do significantly overlap with these indicating sarcopenia, such as reduced grip strength, slow gait speed, and weight loss (14).

Temporal muscle thickness (TMT) has been described as a surrogate parameter to estimate SMM in patients with brain metastases (15). Reduced TMT has been shown to be an independent negative prognostic factor for OS and progression-free survival in patients with progressive glioblastoma (16). Because of its applicability in neuro-oncology without additional examination other than the routine preoperative MRI scan, it was suggested as an attractive and easily assessable parameter for SMM and used further to predict patient outcome (17, 18).

Various TMT cutoff values were described to define sarcopenia. In a study with a large cohort of healthy individuals, it was recommended to be set at 6.3 mm for male and 5.2 mm for female patients (19). The same group found TMT lower than 7.2 mm to be unfavorable for OS and progression-free survival in patients with progressive glioblastoma (16). In another study, TMT was found to be having prognostic value in progressive but not in primary glioblastoma, using a similar cutoff of 7.1 mm (20). On the other hand, some other studies were not able to validate the influence of TMT to OS in glioblastoma patients (21, 22).

We noticed that average TMT in patients of our neuro-oncological database seems to be remarkably thicker than in the previous mentioned studies and their respective cutoff values. This study aimed to evaluate the prognostic relevance of TMT in patients who received first surgical treatment of high-grade glioma in our center. Moreover, we sought to verify the validity of TMT by comparing its usefulness with established functional scoring systems (KPS and CFS) in OS prediction.

## Materials and methods

We included all patients who received first surgical treatment of histologically proven high-grade glioma in our institution in the years of 2015 until 2020.

**Figure 1:**
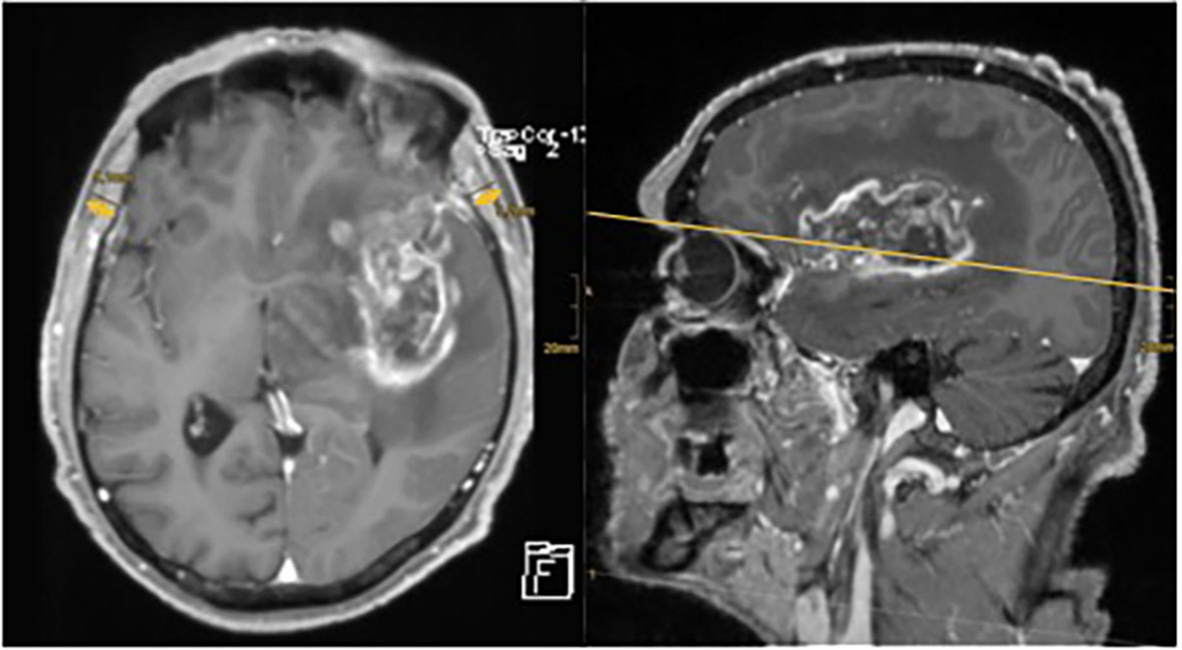
Axial and sagittal MRI images of one of the neuro-oncological database’s patient. There is a large contrast enhancing tumor visible in the left frontotemporal hemisphere. TMT has been measured on the axial plane using the Sylvian fissure and orbital roof as reference points. The arrows indicate the extent of TMT measurement.

To assess TMT, measurements were taken in analogy to previous studies in this field (21–23): in the axial plane of preoperative T1-weighted contrast-enhanced MRI, TMT was measured perpendicularly to the long axis of the temporal muscle—from its inner to outer margin, not including the fascia. A landmark for craniocaudal orientation was the roof of the orbit, and for frontal–occipital orientation, we aimed for the Sylvian fissure (see **Figure 1**). Mean TMT was then calculated by adding left and right side TMT measurements of each patient and dividing the result by two.

**Figure 1** shows an example of TMT measurement in contrast-enhanced axial and sagittal T1-weighted MRI scans of a patient with HGG of our cohort.

Performance status was analyzed in preoperative and follow-up visits 3 to 6 months after surgery using CFS and KPS. Epidemiological and neuropathological data were collected from our neuro-oncological database. WHO grading was confirmed in standardized neuropathological assessment according to the revised 4th WHO classification system of CNS tumors (24), where grade III and IV tumors were considered high-grade and therefore included in the study. Immunohistochemistry (IHC) was applied to reveal R132H mutation of IDH 1, as well as EGFR and nuclear ATRX expression. In patients younger than 40 years of age with IDH wild type, DNA sequencing was added. Presence of 1p/19q-codeletion was analyzed by fluorescence *in situ* hybridization (FISH). DNA sequencing was performed to reveal TERT expression and MGMT promoter methylation status, setting the cutoff at 8%.

Precentral (primary motor cortex) and postcentral gyrus (primary somatosensory cortex), primary visual and auditory cortex, Broca’s and Wernicke’s area, internal capsule, and brainstem were defined as eloquent brain areas.

### Statistical analysis

IBM SPSS Statistics (IBM SPSS Statistics for Mac OS, Version 27.0. Armonk, NY: IBM Corp.) was used to process statistical analysis and graphs. Pearson analysis was performed to detect correlations. Monovariate analysis was supported by *t*-test. Linear regression assessed the influence of multiple variables. Cox regression and Kaplan–Meier processing with logrank test was used to assess OS. Receiver operating characteristic (ROC) analysis and area under the curve (AUC) with consequent Youden index processing were chosen for defining relevant TMT cutoff values. Results with *p* < 0.05 were considered statistically significant.

## Results

### Cohort description

**Figure 2:**
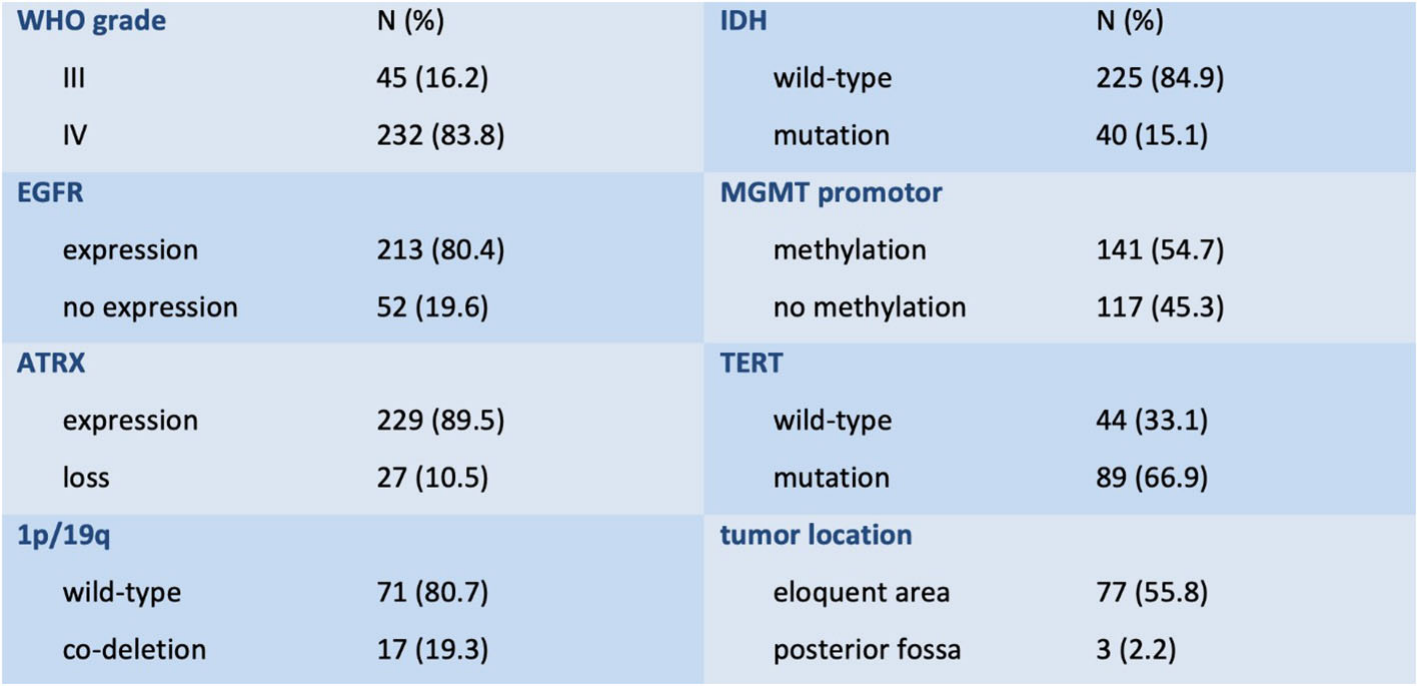
Descriptive data of the study cohort including histological and molecular markers (WHO grade, IDH mutation and MGMT methylation status, EGFR and ATRX expression, 1p/19q co-deletion, and TERT mutation) as well as tumor location in eloquent or non-eloquent brain areas.

A total of 277 patients, 161 (58.1%) men and 116 (41.9%) women, with a mean age of 60 years (95% CI 58–62) were analyzed. Mean TMT was 9.1 mm (95% CI 8.9–9.3) on the right side and 9.0 mm (95% CI 8.8–9.2) on the left side. Regarding functional scores, patients reached a mean KPS of 80 (95% CI 81.3–84.4) preoperatively and 70 (95% CI 64.3–73.2) at follow-up 3 to 6 months after surgery, while mean CFS remained 3 (“managing well”—people whose symptoms are well controlled and who are not regularly active) in both visits (95% CI 2.9–3.3 and 2.9–3.4, respectively). Mean maximal tumor diameter was 4.38 cm (95% CI 4.12–4.64). Of all patients, 35% received total and 50.9% received subtotal resection, while 14.1% underwent tumor biopsy. Subsequently, 75.6% were treated with adjuvant concomitant radio-chemotherapy. More detailed descriptive data are provided in **Figure 2**.

### TMT associations

A significant gender-related difference in TMT could be shown, where mean TMT was 9.8 mm (95% CI 9.5–10.1) in male and 8.1 mm (95% CI 7.8–8.3) in female patients (*p* < 0.001). Mean TMT showed a trend but no significant Pearson correlation with patient age (*p* = 0.07, n.s.). Moreover, there was no significant difference in TMT regarding tumor WHO grade, EGFR expression, ATRX expression, IDH mutation, MGMT promoter methylation, 1p/19q co-deletion, tumor location in the posterior fossa, and maximal tumor diameter (*p* = n.s. for each). However, patients with TERT mutation had significantly thinner TMT (*p* < 0.01), and tumor localization in eloquent brain areas was associated with thicker TMT (*p* < 0.05) in monovariate analysis.

When multifactorial linear regression including age, sex, eloquent location, and TERT mutation was performed, only female gender had a significant influence on mean TMT with measurements being thinner by 1.7 mm (95% CI 0.9–2.5, *p* < 0.001) than in male patients.

### Functional scores *vs*. TMT

To determine a potential interrelation of TMT and functional status of our patients, we analyzed TMT for each step of KPS and CFS scores. **Tables 1** and **2** show TMT measurements per KPS and CFS preoperatively and at the time of follow-up 3–6 months after surgery in patients with HGG. No interrelation in TMT regarding preoperative and follow-up functional scores represented by KPS and CFS could be shown (*p* = n.s. for each).

**Table 1:** Mean TMT for respective KPS scores before surgery and in the follow-up visit.

KPS PO* *(n)*	TMT***	KPS FU** *(n)*	TMT***
10 *(0)*	-_a_	10 *(2)*	8.3 (1.3–15.3)
20 *(0)*	–	20 *(1)*	5.7
30 *(2)*	10.8 (7.3–14.3)	30 *(0)*	–
40 *(1)*	–	40 *(3)*	9.9 (1.4–18.4)
50 *(8)*	9.3 (7.2–11.4)	50 *(9)*	10.5 (8–13)
60 *(15)*	8.1 (7.4–8.8)	60 *(14)*	9.5 (8.5–10.5)
70 *(35)*	8.5 (7.9–9.2)	70 *(28)*	9.2 (8.4–10)
80 *(74)*	9.3 (8.8–9.8)	80 *(26)*	9.4 (8.6–10.1)
90 *(101)*	9.1 (8.8–9.5)	90 *(52)*	8.7 (8.3–9.2)
100 *(41)*	9.1 (8.5–9.6)	100 *(75)*	9.3 (8.8–9.7)

* Preoperative, ** follow-up, *** TMT in mm (95% CI).

a Missing data in TMT columns indicate that there were no patients with the respective KPS score.

**Table 2:** Mean TMT for respective CFS scores before surgery and in the follow-up visit.

CFS PO* *(n)*	TMT***	CFS FU** *(n)*	TMT***
1 *(23)*	9.1 (8.3–10)	1 *(17)*	9.4 (8.4–10.4)
2 *(76)*	9.2 (8.7–9.6)	2 *(79)*	9.1 (8.7–9.5)
3 *(95)*	9.3 (8.8–9.7)	3 *(54)*	9 (8.5–9.5)
4 *(40)*	8.6 (8–9.2)	4 *(23)*	8.9 (8.1–9.7)
5 *(22)*	8.3 (7.4–9.1)	5 *(6)*	10.3 (8.8–11.9)
6 *(9)*	8.9 (7.3–10.5)	6 *(14)*	9.5 (8.5–10.6)
7 *(9)*	9.2 (7.6–10.7)	7 *(10)*	11 (8.7–13.3)
8 *(1)*	6.5	8 *(3)*	7.5 (1.3–13.7)
9 *(0)*	-_a_	9 *(2)*	8.3 (1.3–15.3)

* Preoperative, ** follow-up, *** TMT in mm (95% CI).

a Missing data in TMT columns indicate that there were no patients with the respective CFS score.

Pearson analysis showed a significant correlation of patient age and lower preoperative and follow-up KPS (*r* = −0.23, *p* < 0.001 and *r* = −0.32, *p* < 0.001, correspondingly) as well as patient age and poorer preoperative and follow-up CFS (*r* = 0.24, *p* < 0.001 and *r* = 0.15, *p* < 0.05, correspondingly). Also, a significant correlation of maximal tumor diameter and poor preoperative functional scores was present (*r* = −0.17 for KPS and 0.21 for CFS, *p* < 0.05, correspondingly).

### OS and functional scores

In the mean postoperative follow-up of 20.7 months (95% CI 18.0–23.4), 64.3% of the patients were reported deceased. Estimated OS was 39.9 months (95% CI 33.0–46.8) according to Kaplan–Meier analysis.

Cox regression examining preoperative CFS score and OS showed a hazard ratio (HR) of 1.58 per step of worsening in CFS score (95% CI 1.42–1.75, *p* < 0.001), which means the probability to die within our study follow-up period increased by 58% per additional point in CFS scoring. In patients who were not considered frail preoperatively according to CFS results (CFS 1–4), hazard ratio for death within follow-up was calculated to be 2.7 times lower (HR 3.66, 95% CI 2.49–5.38, *p* < 0.001) than in frail patients (CFS 5–9).

For KPS, the same analysis revealed a reduction of OS by 47% (95% CI 35.2–59.3, *p* < 0.001) per 10 units’ deficit in the preoperative scoring.

### OS and TMT

Cox regression demonstrated no significant influence of TMT on OS (*p* = n.s.). Dividing the patients into subgroups with IDH mutation and IDH wild type, TMT showed no significant effect on OS in either of these subgroups (*p* = n.s.). Functional scores, on the other hand, showed improved OS per point increase in preoperative KPS in IDH wild-type tumors (HR 0.889, 95% CI 0.826–0.956, *p* < 0.01) as well as in IDH mutated tumors (HR 0.913, 95% CI 0.842–0.991, *p* < 0.05). Similar results were presented regarding loss of points in preoperative CFS (HR 2.507, 95% CI 1.537–4.090, *p* < 0.001 in IDH wild type and HR 4.690, 95% CI 1.306–16.842, *p* < 0.05 in IDH mutation per decrease of point in preoperative CFS).

According to recommended gender-specific TMT cutoff values (6.3 mm for male and 5.2 mm for female patients (19)), only three subjects of our population met these criteria: Patient 1 was a 78-year-old woman with status post (st. p.) myocardial infarction, arrhythmia, st. p. bronchial neoplasia, and grade 3 chronic obstructive lung disease who died 5 months after surgery. Patient 2 was a 63-year-old man without any remarkable medical history who is still alive. Patient 3 was an 81-year-old man with known high-grade intestinal neoplasia presenting a large-sized tumor who underwent tumor biopsy and died 3 months later. Owing to the small number of patients in this group, meaningful validation and analytic evaluation for survival estimation could not be performed.

In this study, 45 patients (12 men and 33 women) showed TMT values below the recommended cutoff of 7.2 mm (16). Kaplan–Meier analysis was performed without showing any statistically significant differences in logrank assessment: patients with a TMT ≥ 7.2 mm showed a mean OS of 41.0 months (95% CI 33.4–48.6), while those with TMT < 7.2 mm presented an estimated OS of 26.3 months (95% CI 16.5–36.1, *p* = n.s.).

### ROC/AUC analysis

In this study, the optimal TMT cutoff based on our cohort data to estimate OS was determined by ROC analysis. The optimal TMT cutoff value in our study cohort, defined by Youden index, was 7.6 mm (sensitivity 0.80, specificity 0.36, *p* = n.s.) for 6 months, 7.8 mm (sensitivity 0.74, specificity 0.36, *p* = n.s.) for 12 months, and 8.2 mm (sensitivity 0.71, specificity 0.40, *p* = n.s.) for 24 months OS. Analogically, we determined optimal cutoffs for favorable preoperative KPS and CFS scores according to our patient data, which were KPS ≥ 90 for OS > 6 months (sensitivity 0.64, specificity 0.78, *p* < 0.001) as well as for OS > 12 months (sensitivity 0.70, specificity 0.76, *p* < 0.001) and for OS > 24 months (sensitivity 0.75, specificity 0.60, *p* < 0.001). Scoring CFS ≤ 3 (sensitivity 0.58, specificity 0.82, *p* < 0.001) was favorable for OS > 6 months and OS > 12 months (sensitivity 0.51, specificity 0.86, *p* < 0.001) and CFS ≤ 4 for OS > 24 months (sensitivity 0.20, specificity 0.94, *p* < 0.001).

**Figure 3:**
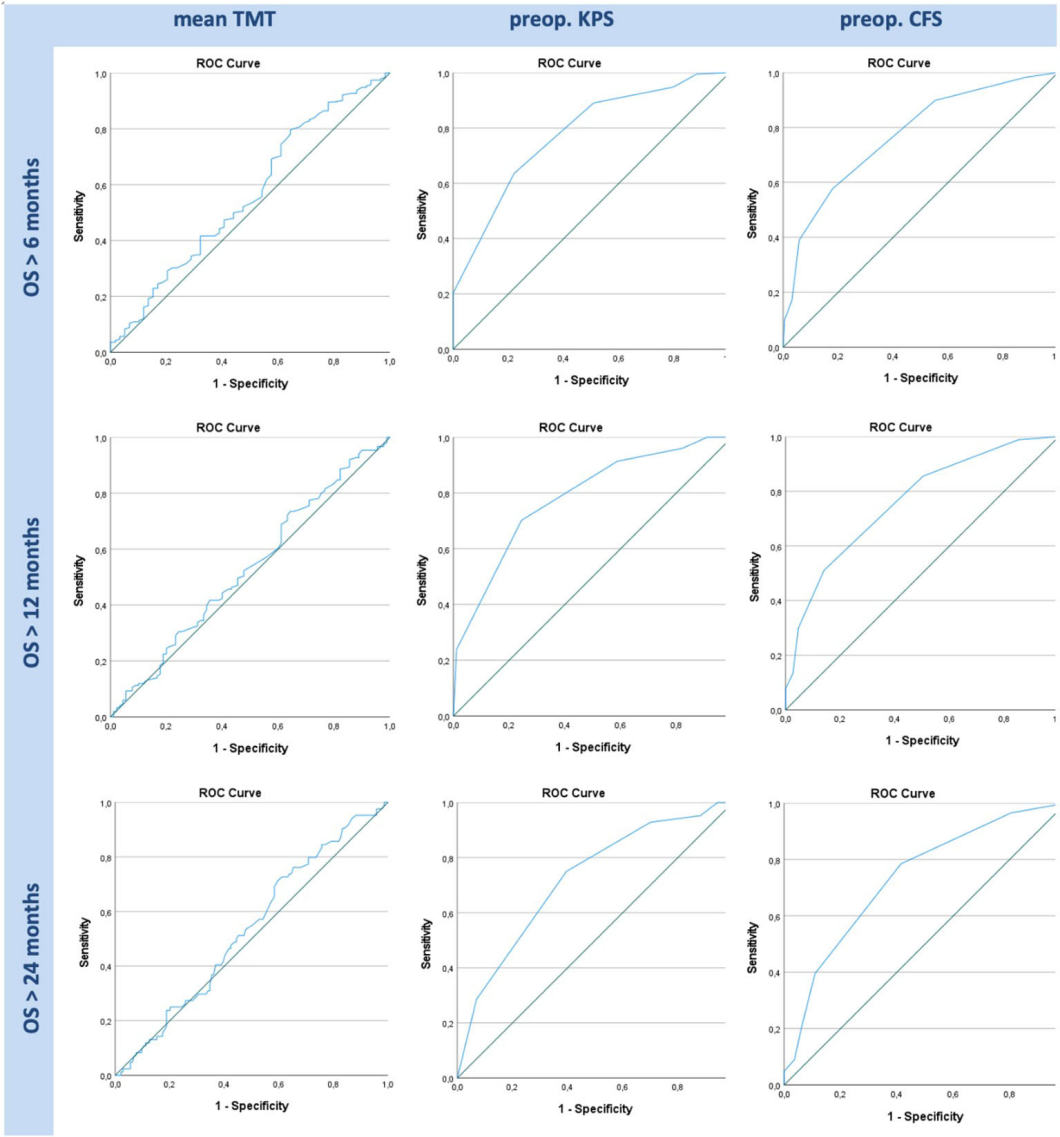
ROC graphs for TMT, preoperative (“preop.”) KPS, and preoperative CFS in OS greater than 6, 12, and 24 months. While KPS and CFS show significant AUC curves in all given time frames, TMT shows no significant data in any of the graphs.

As shown in **Figure 3**, ROC curves for TMT and preoperative functional scores in OS greater than 6, 12, and 24 months demonstrate significant results in KPS (AUC = 0.778, 0.784, and 0.722, correspondingly, *p* < 0.001 for each) and CFS (AUC = 0.772, 0.762, and 0.734, correspondingly, *p* < 0.001 for each). TMT showed no significant AUC values in 6, 12, and 24 months’ follow-up (AUC = 0.562, 0.531, and 0.535, correspondingly, *p* = n.s. for each).

## Discussion

This study on 277 patients could not confirm the usefulness of TMT for prognostic evaluation in high-grade glioma, while functional performance, assessed in CFS and KPS, proved to have a significant impact on OS.

A significant difference in TMT was only shown between male and female patients, where women were proven to have significantly thinner temporal muscles. An analogy to general lower muscle mass in women can be postulated; a study examining over 600 healthy volunteers found significantly higher TMT values in male subjects (19).

On the other hand, investigating the probable causality of lesser TMT with rising patient age showed a trend but no significant correlation.

TMT is indeed an easily accessible and examinable parameter, as almost every neuro-oncological patient receives cranial MRI scans. Therefore, it can certainly be used as a surrogate to determine SMM if this is at interest. In patients with high-grade glioma, however, neurological symptoms leading to hospital admission often arise quite promptly, such as signs of high intracranial pressure or epileptic seizures, and effects on general body condition like sarcopenia probably would not develop noticeably in this short time span.

Previous studies proposed conservative TMT cutoffs, such as 6.3 mm in male and 5.2 mm in female patients, to determine frailty. When trying to apply these recommended cutoff values, only 3 of 277 patients in our cohort qualified as sarcopenic, which makes a meaningful statistical analysis and clinical application impossible. In studies examining TMT in brain metastases, even lower cutoffs were chosen to determine frail patients: 5.9 mm in non-small cell lung cancer patients, 5.8 mm in melanoma patients, and 5.4 mm in breast cancer patients (23, 25). In our study, the optimal cutoff for TMT was 7.6 mm (for OS > 6 months), which is comparable with the proposed 7.2-mm cutoff acquired from the healthy cohort. However, we were not able to validate its influence on survival using our data as there was no statistical significance. Obviously, only relevant systemic disease, often present in patients with metastases, seems to have an effect on TMT.

None of the histologically examined tumor features and molecular markers showed a relevant association with TMT.

We assessed CFS in all of our patients, which is a world-renowned tool to objectively quantify frailty, reaching from very fit to very severely frail patients. It is easily applicable in any pre- or postoperative clinical visit. According to preoperative CFS assessments, 41 patients (15%) from our cohort qualified as frail. At the same time, patient performance, as assessed in performance scales such as CFS and KPS, is known to be highly influential on OS in patients with high-grade brain tumors and metastases. The data in this study confirm the remarkable impact on OS by CFS and KPS scores according to Cox regression.

TMT showed no significant correlation with preoperative or follow-up CFS and KPS scores. Thus, TMT and CFS show no comparable results in detecting frail patients, which raises doubts in the usefulness of the above-mentioned lower TMT cutoffs.

ROC analysis showed similar results in CFS and KPS with a significant impact on 6-, 12-, and 24-month OS, while differences in mean TMT did not entail any effect on OS. Furthermore, non-statistically significant specificity was remarkably poorer (0.36 and 0.40) in TMT, compared to statistically significant preoperative performance scores (0.82, 0.86, and 0.94 for CFS and 0.78, 0.76, and 0.60 for KPS).

We therefore suggest using these functional scores as predicting factors for OS, due to their superiority to TMT, outclassing the latter as a valuable parameter for OS.

In conclusion, TMT does not correlate with functional scores like KPS and CFS or OS in high-grade glioma patients and therefore cannot replace patients’ performance status assessed in a face-to-face visit by the physician. Determining a useful TMT cutoff for OS evaluation remains challenging. The use of TMT cannot be recommended as a single parameter for prediction of OS in patients with high-grade glioma and functional scores such as CFS and KPS outclass the value of TMT by far.

## Data availability statement

The raw data supporting the conclusions of this article will be made available by the authors, without undue reservation.

## Ethics statement

The studies involving humans were approved by ethics committee of the Medical University of Innsbruck (1333/2021). The studies were conducted in accordance with the local legislation and institutional requirements. Written informed consent for participation in this study was provided by the participants’ legal guardians/next of kin.

## Author contributions

Conceptualization, JKl, AK and CF. Methodology, AK, JKl, and CF. Formal analysis, AK. Investigation, JKl, AK, VS, JKe and DP. Resources, AG, CT and CF. Data curation, JKl and AK. Writing—original draft preparation, JKl. Writing—review and editing, AK, CF and CT. Supervision, CF and CT. Project administration, JKl and AK. All authors contributed to the article and approve the submitted version.
